# Enhanced Effects of Chronic Restraint-Induced Psychological Stress on Total Body Fe-Irradiation-Induced Hematopoietic Toxicity in *Trp53*-Heterozygous Mice

**DOI:** 10.3390/life12040565

**Published:** 2022-04-10

**Authors:** Bing Wang, Takanori Katsube, Kaoru Tanaka, Yasuharu Ninomiya, Hirokazu Hirakawa, Cuihua Liu, Kouichi Maruyama, Guillaume Varès, Seiji Kito, Tetsuo Nakajima, Akira Fujimori, Mitsuru Nenoi

**Affiliations:** 1 National Institutes for Quantum Science and Technology, Chiba 263-8555, Japan; katsube.takanori@qst.go.jp (T.K.); tanaka.kaoruqst@gmail.com (K.T.); ninomiya.yasuharu@qst.go.jp (Y.N.); hirakawa.hirokazu@qst.go.jp (H.H.); liu.cuihua@qst.go.jp (C.L.); maruyama.kouichi@qst.go.jp (K.M.); nakajima.tetsuo@qst.go.jp (T.N.); fujimori.akira@qst.go.jp (A.F.); 2 Institut de Radioprotection et de Sûreté Nucléaire, 92260 Fontenay-aux-Roses, France; guillaume.vares@irsn.fr; 3 Center for Animal Research and Education, Nagoya University, Nagoya 464-8601, Japan; sk126@care.nagoya-u.ac.jp

**Keywords:** chronic restraint-induced stress, total-body irradiation, iron-particle radiation, peripheral blood hemogram, bone marrow micronucleated erythrocytes, mouse restraint model

## Abstract

Humans are exposed to both psychological stress (PS) and radiation in some scenarios such as manned deep-space missions. It is of great concern to verify possible enhanced deleterious effects from such concurrent exposure. Pioneer studies showed that chronic restraint-induced PS (CRIPS) could attenuate *Trp53* functions and increase gamma-ray-induced carcinogenesis in *Trp53*-heterozygous mice while CRIPS did not significantly modify the effects on X-ray-induced hematopoietic toxicity in *Trp53* wild-type mice. As high-linear energy transfer (LET) radiation is the most important component of space radiation in causing biological effects, we further investigated the effects of CRIPS on high-LET iron-particle radiation (Fe)-induced hematopoietic toxicity in *Trp53*-heterozygous mice. The results showed that CRIPS alone could hardly induce significant alteration in hematological parameters (peripheral hemogram and micronucleated erythrocytes in bone marrow) while concurrent exposure caused elevated genotoxicity measured as micronucleus incidence in erythrocytes. Particularly, exposure to either CRISP or Fe-particle radiation at a low dose (0.1 Gy) did not induce a marked increase in the micronucleus incidence; however, concurrent exposure caused a significantly higher increase in the micronucleus incidence. These findings indicated that CRIPS could enhance the deleterious effects of high-LET radiation, particularly at a low dose, on the hematopoietic toxicity in *Trp53*-heterozygous mice.

## 1. Introduction

Humans are exposed to both psychological stress (PS) and ionizing radiation (IR) in some scenarios, such as spaceflight crews in deep-space missions, patients in radiotherapy, and residents living in contaminated areas after a nuclear accident. In future human space activities, such as manned missions to lunar and Mars orbit and surface, cosmic radiation, particularly the high atomic number and energy-charged particles of galactic cosmic rays and solar energetic particles, would strongly affect the dosimetry of flight crews [1]. The high atomic number and energy-charged particles are often referred to as densely ionizing or high-linear energy transfer (LET) radiation as they could deposit a large amount of energy along their trajectory, resulting in a high ionization density along the center of the particle path [2]. Although the particles make up only 0.9% of galactic cosmic rays, they will make up 88.1% of the estimated ionizing dose equivalent from exposure to galactic cosmic rays during deep-space travel and thus markedly contribute to the overall biological effects from cosmic radiation [3,4,5]. Among the various components of galactic cosmic rays, iron (Fe) particles are the most densely ionizing and present in relatively large amount [6]. According to estimation and calculation, during manned long-duration deep-space Mars missions, the dose equivalent for even the shortest round-trip would be 0.66 ± 0.12 Sv inside the spacecraft [7]. Almost half of the dose equivalent is high atomic number and energy-charged-particle-derived, with 13% being from Fe particles alone [8,9]. On the other hand, spaceflight crews frequently develop PS during spaceflight due to multiple changes such as the enclosed environment, microgravity, altered light–dark cycles, and the perception of risks of exposure to IR, equipment failure, or fatal mishaps [1]. Compared to the short duration of space flight up to now, namely, the majority was 5 to 7 months long [10], the future missions will take much more time, i.e., missions to Mars, according to conceptual design studies by NASA, may take 800–1000 days, of which approximately 500 days will be spent on the Mars surface [11]. The longer duration of confinement to a spacecraft, encounters with those multiple environmental changes, and isolation from the Earth would increase the risk for the crews to develop PS at a much higher level.

PS occurs when confronting a situation where the demands go beyond the coping resources. As a feeling of strain and pressure, with emotional and physiological reactions, PS could contribute to a number of health consequences, including cancer [12,13,14,15,16]. In humans, epidemiological studies showed that PS could increase the risk of various physiological changes and health consequences from reduced DNA repair capacity, visual impairment, cardiovascular diseases, metabolic syndrome, dementia, and immune-related disorders to cancer development [17,18,19,20,21,22,23,24,25,26,27,28,29]. In animal models, experimental investigations demonstrated that PS could induce sympathetic, neuroendocrinal, and immune responses and activate the hypothalamic–pituitary–adrenal axis and the sympatho–adreno–medullary system, resulting in a variety of adverse health outcomes such as alterations in inflammation and behavior, brain malfunctioning, hematologic abnormality, cardiovascular disease, and tumor growth, progression, and metastasis [25,30,31,32,33,34,35].

Although the effects from exposure to PS on IR-induced health consequences and the mechanisms involved in these outcomes remain largely unknown [30], pioneer studies in mouse chronic-restraint-induced PS (CRIPS) models demonstrated that CRIPS had little modifying effect on X-ray-induced hematological toxicity in peripheral blood and stable type chromosome aberrations in splenocytes of *Trp53*-wild-type mice, but it could attenuate *Trp53* functions, enhance Fe-particle radiation-induced stable-type chromosome aberrations in splenocytes, and increase gamma-ray-induced carcinogenesis in *Trp53*-heterozygous mice [36,37,38,39]. Compared to low-LET photon radiation (i.e., X-rays and gamma-rays), there is much uncertainty in estimates of the biological effectiveness of high-LET particle radiation for the health risk in humans due to the lack of epidemiological data. Further epidemiological studies and experimental investigations in animal models are warranted to explore the biological effects from concurrent exposure to both PS and high-LET particle radiation [1,30]. In this study, a possible modifying effect from exposure to CRIPS on Fe-particle radiation-induced hematopoietic toxicity was investigated in *Trp53*-heterozygous mice. Using micronucleus induction in bone marrow erythrocytes as the index, we aimed to verify if PS could enhance the high-LET Fe-particle radiation-induced genotoxicity. The data obtained are expected to be useful to help better understand the health risk of concurrent exposure to PS and high-LET particle radiation in spaceflight crews on long-duration deep-space missions.

## 2. Materials and Methods

### 2.1. Animals and Experimental Groups

*Trp53*-heterozygous C57BL/6N male mice (BRC NO. 01361, CDB 0001K) [40] were bred in an SPF animal facility at the National Institute of Radiological Sciences (NIRS), National Institutes for Quantum Science and Technology (QST) (Chiba, Japan). At postnatal 4 weeks, animals were transferred to and maintained in a clean conventional temperature- (23 ± 28 °C) and humidity-controlled (50 ± 10%) animal facility at NIRS, QST (Chiba, Japan) under a 12:12 h light–dark schedule (lights on from 7:00 a.m. to 7:00 p.m.). Animals housed in autoclaved aluminum cages with sterilized wood chips were allowed free access to standard laboratory chow (MB-1; Funabashi Farm Co., Funabashi, Japan) and acidified water (pH 3.0 ± 0.2). One or two male mice that were from the same dam and had been together since birth, were housed in the same cage. Animals were acclimatized to the conventional laboratory conditions for 2 weeks. To avoid possible effects from the developmental condition of the animals, at postnatal 6 weeks, any mouse with a significantly different body weight, namely, more or less than the mean ±2 standard deviations (SD), was omitted from this study. Then, selected mice were randomly assigned to 6 experimental groups with 6–12 mice in each group (Table 1): the “control group (C)”, receiving neither chronic restraint nor total body irradiation (TBI) with Fe-particle radiation (Fe); the “irradiation group exposed to 0.1 Gy Fe-TBI (Fe0.1)”, receiving only Fe-TBI at 0.1 Gy; the “irradiation group exposed to 2.0 Gy Fe-TBI (Fe2.0)”, receiving only 2.0 Gy Fe-TBI; the “CRIPS group (S)”, receiving only chronic restraint; the “chronic restraint and irradiation group exposed to 0.1 Gy Fe-TBI (S + Fe0.1)”, receiving both chronic restraint and 0.1 Gy Fe-TBI; and the “chronic restraint and irradiation group exposed to 2.0 Gy (S + Fe2.0)”, receiving both chronic restraint and 2.0 Gy Fe-TBI. One or two male mice from the same dam were housed in the same cage during the 28-day restraint regimen.

### 2.2. Mouse Model for Chronic-Restraint-Induced Psychological Stress

A well-established mouse model for CRIPS [38] with minor modification [36,37,39] was adopted and applied to the present work. In brief, the mouse restraint system (Flat Bottom Rodent Holder, RSTR541, Kent Scientific Co., Torrington, USA) was used for chronic periodic restraint on a daily basis of 6 h for 28 consecutive days. Individual mice aged 6 weeks were placed in the strainer, and the restrained mice were maintained horizontally in their home cage during the 6-h restraint session (9:30 a.m. to 3:30 p.m.) daily; then, the animals were released into the same cage and allowed to access food and water during the free session (3:30 p.m. to 9:30 a.m.). The animals in the C, Fe0.1, and Fe2.0 groups received no restraint but abstained from food and water while the animals in S, S + Fe0.1, and S + Fe2.0 groups underwent the 6-h restraint session each day.

### 2.3. Irradiation

Iron particles were generated and accelerated by a synchrotron, the Heavy Ion Medical Accelerator in Chiba (HIMAC), QST, Japan. The monoenergetic ion beams having 500 MeV/nucleon of initial energy were expanded by wobbler magnets to a 10 cm irradiation field with homogeneous irradiation dose. Animals were irradiated at the entrance (plateau) region of the beams. The dose-averaged LET value calculated by the Monte-Carlo simulation was 200 ± 20 KeV/μm. In the early morning (3:30–04:30 a.m. or 6:00–7:00 a.m.), on day 8 of the 28-day restraint regimen, TBI with a dose of 0.1 or 2.0 Gy was performed at a dose rate of about 0.08–0.09 Gy/min or 1.0–2.0 Gy/min, respectively. The reasons for choosing the doses are that the effects from a low dose at 0.1 Gy was of great concern from a practical point of view, and a high dose at 2.0 Gy was used as a positive control as it could induce hematopoietic toxicity in mice. For receiving TBI, the mice were held in a Lucite columnar container, which had an outer diameter of 10 cm and 3 individual cells of the same size (each mouse in each cell). The mice were in an air-breathing condition (there were 6 holes 5 mm in diameter in the wall of each cell). The containers were set on the beam track, and the focused 10 cm diameter ion beam was delivered to the animals at room temperature without anesthesia. 

### 2.4. Evaluation of Physiological Conditions

All animals were checked daily for their wellbeing and any deaths. Changes in body weight gain as an index was assessed to evaluate physiological effects from exposure to CRIPS and/or Fe-TBI. For monitoring body weight gain, all the animals were weighed daily.

### 2.5. Assessment of Peripheral Hemogram

At the end of the restraint regimen, the animals were anesthetized by inhalation of gaseous isoflurane (2-chloro-2-(difluoromethoxy)-1,1,1-trifluoro-ethane) (ISOFLURANE Inhalation Solution, Pfizer, Tokyo, Japan). For analysis of hemogram, the peripheral blood was collected from a femoral artery with a heparinized syringe in vacutainer blood collection tubes containing EDTA (Venoject II, Terumo Co., Tokyo, Japan), and the animals were killed by cervical dislocation. The blood samples were immediately subjected to a differential blood cell count and hemoglobin concentration measurement using a blood cell differential automatic analyzer (SYSMEX K-4500, Sysmex Corporation, Kobe, Japan).

### 2.6. Micronucleus Assay

Micronucleus is one of the structural aberrations caused by improperly repaired DNA breaks and chromosome segregation errors [41,42]. As a responsive test, the micronucleus assay is one of the most popular methods to assess the genotoxicity of different factors, including CRIPS and IR [36,43]. This technique was used in the present work to study the effects on induction of hematopoietic toxicity by exposure to CRIPS and/or Fe-TIB in bone morrow erythrocytes in mice. Bone marrow was collected from both femurs. Then, bone marrow smears were prepared and processed for the enumeration of micronucleated polychromatic erythrocytes (MNPCEs) and micronucleated normochromatic erythrocytes (MNNCEs). The slides were coded to avoid observer bias. The micronuclei were scored using a light microscope at a magnification of 1000×. At least 15600 PCEs and NCEs per mouse were counted. In addition, the percentage of PCEs to the sum of PCEs and NCEs were used as an indicator for evaluating bone marrow erythrocyte proliferation (erythropoiesis).

### 2.7. Statistical Analysis

Statistical evaluation of the data was conducted with Student’s t-test for the difference between the mean  ±  standard deviation from two groups. Statistical significance was assigned to *p* < 0.05. Because the current study involves exploratory research on radiation risk, β error, including overlooking risk, is more harmful than an α error, and there is no point in keeping the α error at a strictly nominal level. Therefore, no α adjustment for multiple testing was conducted [44]. 

### 2.8. Ethics Approval and Disclosure

All experimental protocols involving mice were reviewed and approved by The Institutional Animal Care and Use Committee of the NIRS, QST, Chiba, Japan. The experiments were performed in strict accordance with the NIRS Guidelines for the Care and Use of Laboratory Animals.

An earlier version of this work was presented as a poster at The 64th Annual Meeting of the Radiation Research Society, Chicago, IL, USA.

## 3. Results

### 3.1. General Physiological Conditions

The animals looked exhausted to some extent just after restraint, while no significant difference in physiological appearance (i.e., coat and hair glossiness) or behavior were observed about 3 h after being released. No fighting was observed among mice in any of the cages. No mortality occurred throughout the whole monitoring period.

### 3.2. Verification of the CRIPS Model

**Figure 1:**
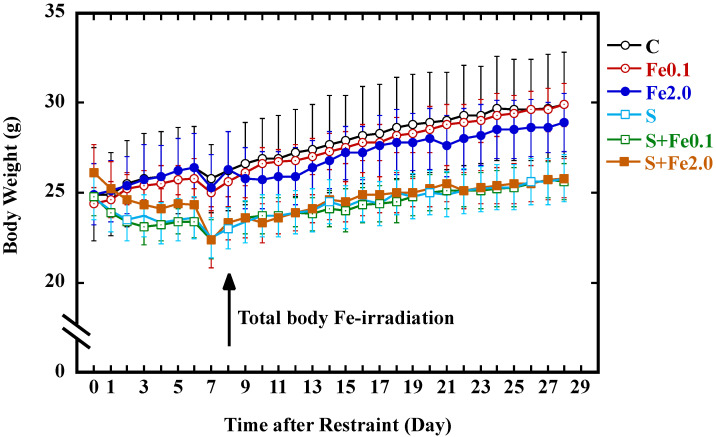
Effect of CRIPS and TBI on body weight gain of mice. Group mean ± SD levels of the control group (C, open circle in black), the TBI with 0.1 Gy group (Fe0.1, midpoint circle in red), the TBI with 2.0 Gy group (Fe2.0, solid circle in blue), the CRIPS group (S, open square in light blue), the CRIPS and TBI with 0.1 Gy group (S + Fe0.1, midpoint square in green), and the CRIPS and TBI with 2.0 Gy group (S + Fe2.0, solid square in brown). Note: the body weight was measured each day just before starting restraint except for on the 7th day and the 28th day when the weighing was performed just after restraint.

The restraint treatments used in this work were shown previously to induce a stress response sufficient to significantly affect several immunological parameters [38]. Under the experimental setup in the present study, reproducibility of this CRIPS model in *Trp53*-heterogygous male mice was verified using endpoints including changes in body weight gain and immune organs (i.e., thymus and spleen) and alterations in the concentration of corticosterone in urine. Significantly reduced body weight gain induced by CRIPS appeared one day after onset of the restraint, and this phenomenon was observed throughout the experiment in the mice of the S, S + Fe0.1, and S + Fe2.0 groups that received the restraint (Figure 1). In addition, a marked decrease in the number of splenocytes per spleen (1.14 ± 0.15 (×10^8^) cells in group C vs. 0.83 ± 0.11 (×10^8^) cells in group S) and a significant increase in concentration of stress-related hormone corticosterone (32.8 ± 19.4 ng/mL in group C vs 91.2 ± 29.6 ng/mL in group S) were also detected. Details for these stress-related changes were reported [39] or will be reported elsewhere. These results clearly confirmed that the establishment of the CRIPS model under our experimental setup was successful.

### 3.3. Change in Body Weight Gain

The effects from CRIPS and TBI with Fe-particle radiation on the body weight gain of mice are shown in Figure 1. Regardless of both the treatments (CRIPS and TBI) and the extent of body weight gain, the body weight of the animals in all groups in general showed a tendency to increase throughout the whole monitoring period. On the other hand, CRIPS, rather than TBI, showed a big impact on inhibition of body weight gain. As a fact, significant decreases in body weight were observed in all groups one day after onset of the restraint (*p* < 0.05): Just before restraint onset (day 0), the body weights (g) for the C, Fe0.1, Fe2.0, S, S + Fe0.1, and S + Fe2.0 groups were 24.9 ± 2.6, 24.4 ± 0.9, 24.9 ± 1.7, 24.7 ± 0.9, 24.8 ± 1.1 and 26.1 ± 1.6, respectively; on the second day before restraint (day 2), the body weights were 25.5 ± 2.4, 25.2 ± 0.9, 25.4 ± 1.6, 23.5 ± 1.2, 23.4 ± 1.1, and 24.6 ± 1.4, respectively. The difference in body weight between the animals receiving the restraint and the animals receiving no restraint appeared to be the most significant (*p* < 0.01) since, on the seventh day after restraint, the body weights (g) for the C, Fe0.1, Fe2.0, S, S + Fe0.1, and S + Fe2.0 groups were 25.8 ± 1.9, 25.0 ± 0.8, 25.3 ± 1.8, 22.5 ± 1.1, 22.4 ± 1.1, and 22.4 ± 1.6, respectively. Low body weight gain continued in animals receiving the restraint regardless of TBI to the end of the experiment. The body weights after restraint on the last day of the experiment for the C, Fe0.1, Fe2.0, S, S + Fe0.1, and S + Fe2.0 groups were 29.9 ± 2.9, 29.9 ± 1.2, 28.9 ± 1.6, 25.8 ± 1.2, 25.6 ± 1.0, and 25.8 ± 1.1, respectively. These results indicated that TBI with 0.1 Gy Fe-particle radiation did not show any marked effects on body weight gain while TBI with 2.0 Gy Fe-particle radiation could induce a reduction in the body weight gain to a certain, but not significant, extent; however, exposure to CRIPS was more effective in terms of causing a continuous reduction in body weight gain in mice.

### 3.4. Hematological Abnormality in the Peripheral Hemogram

**Figure 2:**
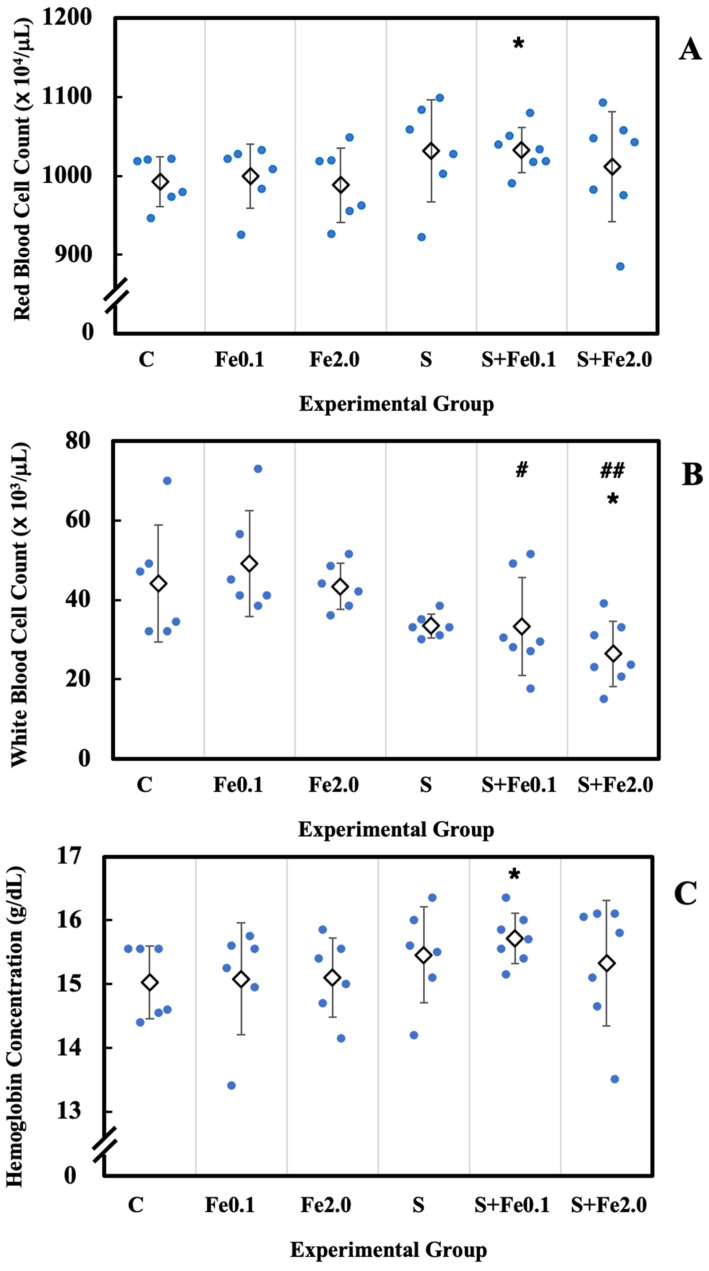

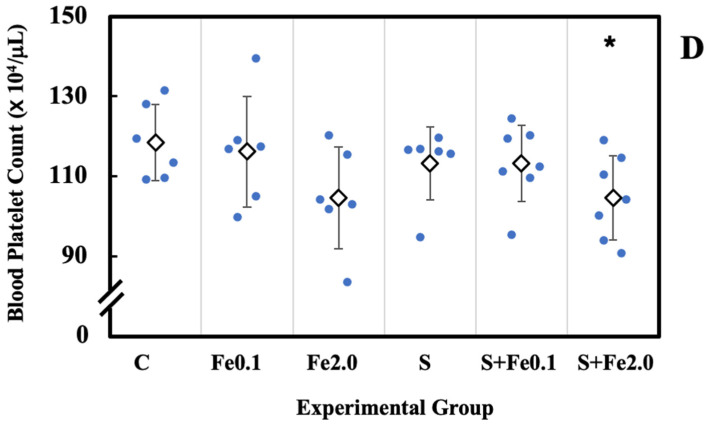
Alteration in peripheral hemogram. Hematological abnormality in the peripheral blood was analyzed using red blood cell count (**A**), white blood count (**B**), hemoglobin concentration (**C**), and blood platelet count (**D**) as indices. One asterisk (*) stands for statistical significance at *p* < 0.05 between the nontreated control group and the treated group. One hash mark (#) and two hash marks (##) stands for statistical significance at *p* < 0.05 and *p* < 0.01, respectively, between the Fe0.1 group and the S + Fe0.1 group and between the Fe2.0 group and the S + Fe2.0 group.

CRIPS- and TBI-induced hematopoietic toxicity measured as alterations in the peripheral hemogram was studied at the end of the experiment. As shown in Figure 2, mice subjected to CRIPS alone (Group S) displayed a slight increase in both red blood cell count and hemoglobin concentration. Although the change is not of statistical significance, CRIPS appeared to cause a decrease in white blood cell count and a slight decrease in blood platelet count. On the other hand, TBI with 0.1 Gy or 2.0 Gy Fe-particle radiation induced alterations in white blood cell count and blood platelet count, but neither of them were of statistical significance. For concurrent exposure to both CRIPS and TBI, the treatment caused a marked increase in both red blood cell count and hemoglobin concentration in the S + Fe0.1 group, and a significant decrease in the white blood cell count in the S + Fe2.0 group. A significantly decreased white blood cell count was also observed in the S + Fe0.1 group and the S + Fe2.0 group when compared to their counterpart receiving only Fe-TBI. These results indicated that CRIPS alone or IR alone could cause, to a certain extent, alterations in peripheral hemogram. However, concurrent exposure to both CRIPS and IR showed a big impact on the induction of hematological abnormality in the peripheral hemogram.

### 3.5. Residual Damage in Bone Marrow Erythrocytes

**Figure 3:**
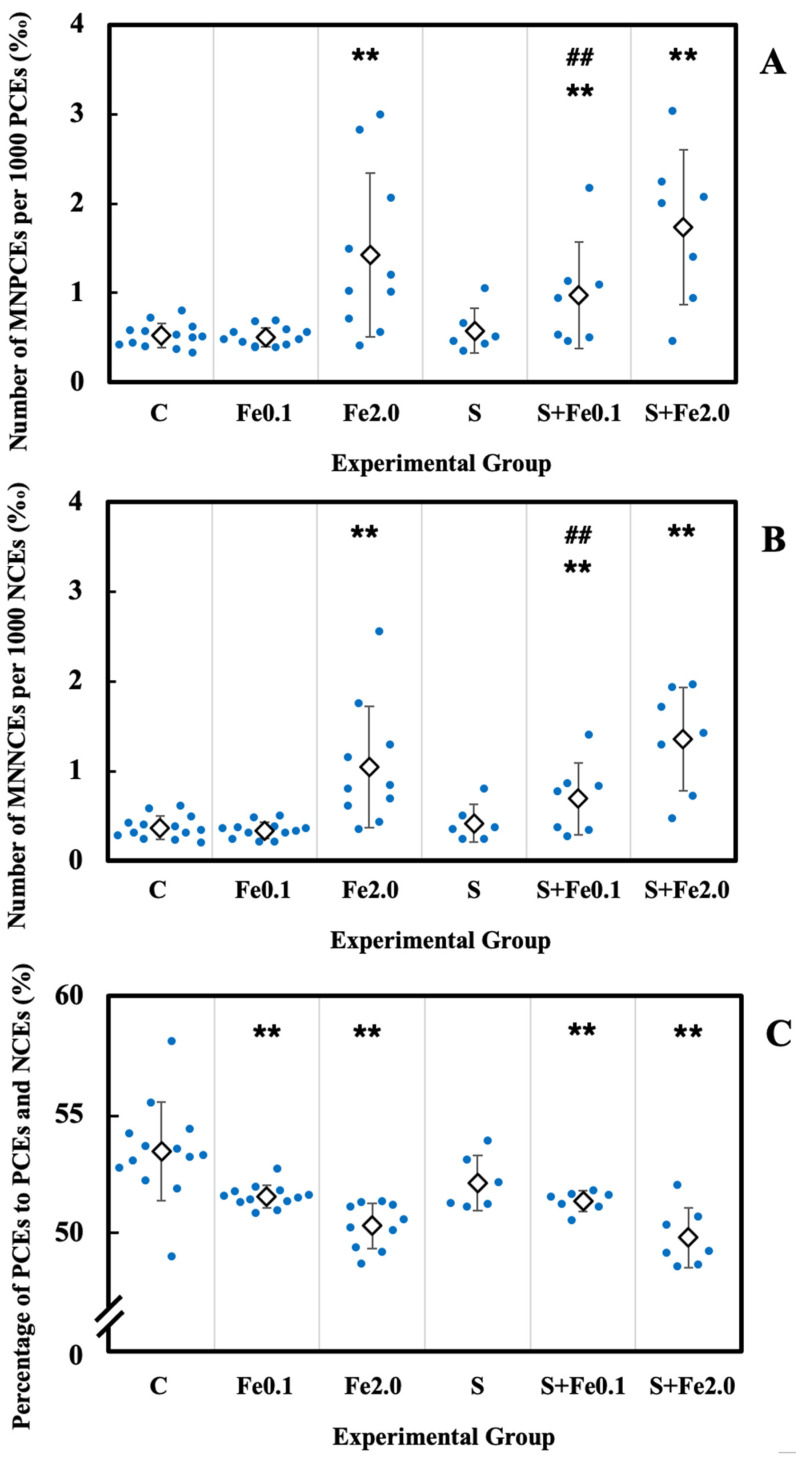
Effect of CRIPS and TBI on the bone marrow erythrocytes of mice. Group mean ± SD of the number of MNPCEs per 1000 PCEs (**A**), the number of MNNCEs per 1000 NCEs (**B**), and the percentage of PCEs to the sum of PCEs and NCEs (**C**). Two asterisks (**) stand for statistical significance at *p* < 0.05 and *p* < 0.01, between the nontreated control group and the treated group. Two hash marks (##) indicates statistical significance at *p* < 0.01 between the Fe0.1 group and the S + Fe0.1 group.

The cytotoxicity and genotoxicity induced by CRIPS and TBI were studied in the bone marrow erythrocytes of the animals. For genotoxic assessment, the incidence of micronucleated erythrocytes including MNPCEs and MNNCEs was used, and its increase indicated elevated genotoxicity. For the evaluation of erythrocyte proliferation, the number of PCEs expressed as a percentage of the sum of PCEs and NCEs was used as an indicator of bone marrow proliferation in the erythroid lineage, and its decrease indicated raised cytotoxicity [45]. As shown in Figure 3, CRIPS alone (Group S) showed no significant effect on the incidence of MNPCEs in PCEs, the occurrence of MNNCEs in NCEs, and the percentage of PCEs with respect to the sum of PCEs and NCEs when compared with that in the control group (Group C). On the other hand, TBI with 2.0 Gy Fe-particle radiation alone resulted in a marked increased incidence of both MNPCEs and MNNCEs (*p* < 0.01) and a significant decreased percentage of PCEs to the sum of PCEs and NCEs (*p* < 0.01). Even at a dose of 0.1 Gy, TBI could also cause significant a decrease in the percentage of PCEs to the sum of PCEs and NCEs (*p* < 0.01). Concurrent exposure to both CRIPS and TBI at 0.1 Gy Fe-particle radiation also markedly induced a reduction in the percentage of PCEs to the sum of PCEs and NCEs. Of note, for the induction of genotoxicity measured as incidences of MNPCEs and MNNCEs, exposure to either CRIPS (Group S) or TBI at a dose of 0.1 Gy (Group Fe0.1) did not lead to any significant effect; however, concurrent exposure to both (Group S + Fe0.1) could cause a marked increase in the incidences of MNPCEs and MNNCEs. In addition, although it was of no statistical significance, concurrent exposure to CRIPS and TBI with 2.0 Gy Fe-particle radiation could also slightly increase the induction of MNPCEs and MNNCEs when compared to that induced by exposure to TBI alone. These results indicated that CRIPS alone did not show any significant impact on the induction of cytotoxicity or genotoxicity in the bone marrow erythrocytes, nor the TBI with Fe-particle radiation at a low dose on induction of genotoxicity. However, CRIPS showed a markedly enhanced effect on low-dose Fe-particle radiation-induced cytotoxicity and genotoxicity in bone marrow erythrocytes.

## 4. Discussion

Epidemiological studies in humans and experimental investigations in animal models clearly show that both PS and IR could induce harmful effects on health outcomes. For example, PS could influence the hematopoietic and immune systems, such as by inducing hematopoietic toxicity manifesting as alterations in hematogenesis, peripheral hemogram (i.e., cell count, change of lymphocyte subsets, and distribution), gene expression of white blood cells, and impairment of erythrocyte and T-lymphocyte immune function [46,47,48,49,50,51,52,53]. In addition, PS is also a well-accepted risk factor in cancer initiation and progression as PS-induced inflammation and immune dysfunction could cause metabolism disorder and difficulties in maintaining homeostasis; increase susceptibility to cancer; and promote cancer initiation, progression, and metastasis [54,55,56,57,58,59]. Exposure to IR at low doses could also lead to various health consequences, such as genetic and epigenetic changes associated with a range of physiological disturbances [60,61,62]. As human activities under certain conditions such as crewed space exploration would unavoidably encounter exposure to PS and high-LET IR, and high-LET IR could cause differential biological effects compared to that caused by low-LET IR [63,64], it is of great concern to verify if any enhanced biological effects could be induced from concurrent exposure to both PS and high-LET IR at low doses.

On the other hand, it is known that genetic background can directly influence cellular responses to IR [65]. As “the guardian of the genome”, the *Trp53* gene responds to myriad stresses, including PS and IR, and plays a critical role in conserving genome stability by preventing mutation [66,67,68]. Loss of *Trp53* function due to mutation and deletion could alter susceptibility to stresses and compromise tumor suppression [68,69,70]. Experimental studies show that *Trp53*-heterozygous mice develop tumors induced by genotoxic carcinogens with a shorter latency than their wild-type counterparts and have been proposed as an alternate animal model for carcinogenicity testing. *Trp53*-heterozygous mice are also more sensitive to the short-term effects of genotoxic agents and manifest a haploinsufficiency phenotype that could contribute to the higher tumor susceptibility. For hematopoietic system, exquisite regulation of *Trp53* activity is critical for maintaining homeostasis under normal and stress conditions, and loss of *Trp53* function could promote leukemia and lymphoma development in humans and mice [71]. In contrast to *Trp53*-independent apoptosis induced by high-LET IR in cultured cells *in vitro* [72], cell death in *vivo* induced by high-LET radiation is at least partially *Trp53*-dependent [73]. IR-induced hematopoietic stem cell apoptosis is via the *Trp53*-*Puma* pathway [74]. As a fact, in mouse bone marrow cells, both *Trp53*-dependent and -independent apoptosis occur after IR exposure, and *Trp53* null cells are more resistant to the induction of apoptosis by IR than *Trp53*-heterozygous cells and *Trp53* wild-type cells [75]. High-LET particle radiation caused higher levels of cytogenetic damage manifesting as a significant increase in micronucleus frequency in hematopoietic stem and progenitor cells when compared to photon radiation at the same dose (0.5 or 1 Gy), contributing to genomic instability and a higher risk of leukemogenesis [76]. As *Trp53*-heterozygous mice are of significant use for the prospective identification of genotoxic carcinogens that bear potential risk to human health [77,78], in the present work, *Trp53*-heterozygous mice are used to verify the effects from concurrent exposure to PS and high-LET Fe-particle radiation on induction of hematopoietic toxicity.

In humans, it is reported that PS could induce hematological changes in peripheral hemogram [79,80], for example, increased red blood cell count and hemoglobin concentration [79]. Studies also show reduced functioning of immune cells, elevated plasma cytokine profiles and persistent inflammation in most crews during spaceflight [81,82,83,84,85]. In this work, the results showed that CRIPS could induce an increased tendency in red blood cell count regardless of exposure to Fe-particle radiation. Concurrent exposure to CRIPS and Fe-TBI (0.1 Gy) could even lead to a statistically significant increase. Interestingly, a CRIPS-induced increase in red blood count was not observed in our previous study using *Trp53* wild-type mice [36]. As *Trp53* plays a critical role in regulation of oxygen and redox homeostasis [86], elevated red blood count may point towards alterations in cellular metabolism under CRIPS in *Trp53*-heterozygous mice [87]. In addition, hemoconcentration could also hasten procoagulatory status [88], playing a critical role in stress-induced pathogenesis of various health consequences such as cardiovascular disease and brain malfunction [33]. For the effects from PS on induction of genotoxicity by Fe-particle radiation, exposure to either CRISP or Fe-particle radiation (0.1 Gy) did not induce a marked increase in the incidence of micronucleated erythrocytes, while concurrent exposure to CRISP and Fe-particle radiation (0.1 Gy) caused significantly higher increase in the incidence. Of note, in *Trp53* wild-type C57BL/6 J male mice, previous investigations using the same CRIPS model showed that CRIPS alone did not cause increased chromosomal aberrations in splenocytes and elevated micronuclei in bone marrow erythrocytes. CRIPS did not appear to synergize with the TBI (4.0 Gy X-ray radiation) clastogenicity and genotoxicity [36,37]. However, in splenocytes collected from the same *Trp53*-heterozygous C57BL/6N male mice in the present work, our recent work demonstrated that neither CRIPS nor TBI (0.1 Gy Fe-particle radiation) alone induced any increase in the frequency of aberrant chromosomes while concurrent exposure resulted in a statistically significant increase in the frequency of chromosomal exchanges [39]. Interestingly, in testicular tissues that were collected from the same animals in the present work, studies showed that CRISP did not significantly exacerbate Fe-particle radiation-induced testicular toxicity measured as apoptosis and structure damage [89]. In addition, in kidneys that were also collected from these mice, no enhanced effects from CRISP on Fe-TBI-induced renal damage were observed (a paper under submission). In fact, functional *Trp53* dynamics vary between tissues and are frequently implicated in contributing to radiation sensitivity through activating subsets of target genes to carry out cell fates (i.e., apoptosis, cell cycle arrest, and DNA repair) [90,91,92,93]. Together, these findings also suggest that studies on concurrent exposure to PS and IR should be carried out using different endpoints in different tissues and in animals with different genetic background.

Experimental investigations using PS models in laboratory animals are of great concern as the findings will not only support but also potentially improve the translational research from animal models to practical trials. Further molecular, cellular, and physiological mechanism studies are expected to understand the biological basis, enhance the translational approach, bridge the gap between animals and humans, potentialize clinically applicable stress-oriented strategies, and help therapeutic approaches in prevention, intervention, and treatment of the detrimental effects from exposure to PS. Health studies in humans under space analogue conditions showed a significant inter-individual variability in response to environmental stressors. For future manned deep-space missions, personalized countermeasures based on genetic background and molecular and epigenetic alterations for flight crews are imperative to minimize detrimental effects from concurrent exposure to multiple physical, physiological, and psychological stresses. On the other hand, advances in space radiation biology studies could also be translated to practical applications to humans on the Earth, and vice versa [1,94,95].

## 5. Conclusions

In the CRIPS model using *Trp53*-heterozygous mice, exposure to either CRISP or high-LET Fe-particle radiation at a dose of 0.1 Gy did not induce a marked increase in the incidence of micronucleated erythrocytes in bone marrow, while concurrent exposure could cause a significant increase in the incidence. These findings indicate that CRIPS could enhance the effects of high-LET particle radiation on the induction of hematopoietic toxicity in *Trp53*-heterozygous mice. These findings also suggest critical effects from cell-intrinsic factors (genetic background) and cell-extrinsic factors (alterations in microenvironment due to PS) on the induction of hematopoietic toxicity by IR, raising awareness of the enhanced deleterious effects of PS on genotoxicity induced by high-LET particle radiation.

## Figures and Tables

**Table 1:** Experimental Group.

Abbreviation	Treatment
C	The control group, receiving neither chronic restraint nor total body irradiation (TBI) with Fe-particle radiation (Fe)
Fe0.1	The irradiation group exposed to 0.1 Gy Fe-TBI, receiving only Fe-TBI at 0.1 Gy
Fe2.0	The irradiation group exposed to 2.0 Gy Fe-TBI, receiving only 2.0 Gy Fe-TBI
S	The chronic restraint-induced psychological stress (CRIPS) group, receiving only chronic restraint
S + Fe0.1	The chronic restraint and irradiation group exposed to 0.1 Gy Fe-TBI, receiving both chronic restraint and 0.1 Gy Fe-TBI
S + Fe2.0	The chronic restraint and irradiation group exposed to 2.0 Gy Fe-TBI, receiving both chronic restraint and 2.0 Gy Fe-TBI

## Data Availability

Data supporting the findings of the present study are available within the article.
